# When Are Depolarizing GABAergic Responses Excitatory?

**DOI:** 10.3389/fnmol.2021.747835

**Published:** 2021-11-24

**Authors:** Werner Kilb

**Affiliations:** Institute of Physiology, University Medical Center of the Johannes Gutenberg-University Mainz, Mainz, Germany

**Keywords:** chloride homeostasis, NKCC1, KCC2, SLC12A2, SLC12A5, gaba receptor, neuronal development

## Abstract

The membrane responses upon activation of GABA(A) receptors critically depend on the intracellular Cl^−^ concentration ([Cl^−^]_i_), which is maintained by a set of transmembrane transporters for Cl^−^. During neuronal development, but also under several pathophysiological conditions, the prevailing expression of the Cl^−^ loader NKCC1 and the low expression of the Cl^−^ extruder KCC2 causes elevated [Cl^−^]_i_, which result in depolarizing GABAergic membrane responses. However, depolarizing GABAergic responses are not necessarily excitatory, as GABA(A) receptors also reduces the input resistance of neurons and thereby shunt excitatory inputs. To summarize our knowledge on the effect of depolarizing GABA responses on neuronal excitability, this review discusses theoretical considerations and experimental studies illustrating the relation between GABA conductances, GABA reversal potential and neuronal excitability. In addition, evidences for the complex spatiotemporal interaction between depolarizing GABAergic and glutamatergic inputs are described. Moreover, mechanisms that influence [Cl^−^]_i_ beyond the expression of Cl^−^ transporters are presented. And finally, several *in vitro* and *in vivo* studies that directly investigated whether GABA mediates excitation or inhibition during early developmental stages are summarized. In summary, these theoretical considerations and experimental evidences suggest that GABA can act as inhibitory neurotransmitter even under conditions that maintain substantial depolarizing membrane responses.

## Introduction

About 30–40 years ago it was first published that GABA_A_ receptors can mediate depolarizing and even excitatory membrane responses in the immature brain (Mueller et al., 1983; Ben-Ari et al., 1989; Luhmann and Prince, 1991), in contrast to the general textbook knowledge that GABA mediates inhibitory and mostly hyperpolarizing neurotransmission in the CNS. In the following decades, it has been shown that depolarizing GABAergic membrane responses play an essential role for cortical development (Ben-Ari, 2002; Owens and Kriegstein, 2002; Kirmse and Holthoff, 2020) and that such depolarizing GABAergic responses can be re-attained under pathophysiological conditions like trauma, stroke or epilepsy (Jaenisch et al., 2010; Dzhala et al., 2012; Kaila et al., 2014; Liu et al., 2020).

The main molecular mechanisms underlying these depolarizing membrane responses have in the meantime been unraveled (Blaesse et al., 2009; Loscher et al., 2013; Watanabe and Fukuda, 2015; Virtanen et al., 2020) and the existence of depolarizing GABAergic responses had been demonstrated *in vivo* (Kirmse et al., 2015; Valeeva et al., 2016; Murata and Colonnese, 2020). Thus evidences indicate that the direction of GABAergic membrane responses shows a striking modification during neurodevelopment and under pathophysiological conditions, a process termed “GABA-shift”. Regarding the physiological consequences of this “GABA-shift” it is, however, important to consider that depolarizing GABAergic responses can mediate inhibition already during early postnatal development (Khalilov et al., 1999; Kolbaev et al., 2011a; Kirmse et al., 2015; Valeeva et al., 2016) and that inhibitory responses in the adult CNS can be accompanied by GABAergic depolarizations (Andersen et al., 1980; Misgeld et al., 1982; Staley and Mody, 1992). To provide a current concept of the functional impact of depolarizing GABAergic responses, I summarize in this review theoretical considerations and experimental studies that illustrate how the relation between GABA conductances, GABA reversal potential and membrane potential changes determines the impact of GABA on neuronal excitability. In addition, I review studies that directly investigated whether GABA mediates excitation during early developmental stages.

## Cl^−^ and Hco_3_^−^ Set The Pace for The Effects of GABA_A_ Receptors

**Figure 1:**
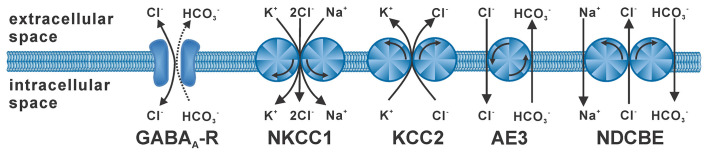
Stoichiometry and typical operation of secondary active Cl^−^ transporters. The GABA_A_ receptor (GABA_A_-R) mediates mainly Cl^−^ fluxes and to a lesser extent HCO_3_^−^ fluxes. NKCC1 mediates uptake of two Cl^−^ ions with of one K^+^ and one Na^+^ ion. KCC2 mediates extrusion of one Cl^−^ with one K^+^ ion. The anion-exchanger (AE3) is supposed to mediate uptake of one Cl^−^ ion in antiport with one HCO_3_^−^ ion. The Na^+^-dependent Cl^−^/HCO_3_^−^ exchanger (NDCBE) utilizes the Na^+^ gradient to extrude Cl^−^ ions.

The flux of the hydrophilic Cl^−^ ions across the hydrophobic plasma membrane occurs exclusively *via* integral membrane proteins that mediate Cl^−^ transport. Passive Cl^−^ fluxes are mediated by a heterogeneous set of anion channels that are more or less specific for Cl^−^ ions (Duran et al., 2010; Jentsch and Pusch, 2018), including the GABA_A_ receptor (Farrant and Kaila, 2007). The passive Cl^−^ fluxes through these anion channels follow the electromotive force for Cl^−^ ions (EMF_Cl_), which depends on the difference between the Cl^−^ equilibrium potential (E_Cl_) and the membrane potential (E_m_). In consequence, hyperpolarizing or depolarizing GABAergic responses require that the intracellular Cl^−^ concentration ([Cl^−^]_i_) is not in an equilibrium state. Active transmembrane transport is required for accumulation or depletion of Cl^−^ from cells (Huebner and Holthoff, 2013; Kaila et al., 2014). In the absence of such active transport processes [Cl^−^]_i_ follows a passive distribution, which due to the negative E_m_ is set at low millimolar concentrations under physiological conditions (given by the Nernst equation). Either primary active Cl^−^ transport, *via* an ATP-dependent Cl^−^ pump, or secondary active transport, coupling Cl^−^ transport to the transport of another ions along their gradient, is required to obtain [Cl^−^]_i_ below or above this passive distribution. The major proteins that mediate secondary active transmembrane Cl^−^ transport are Na^+^-dependent K^+^/Cl^−^-cotransporters (NKCC), K^+^/Cl^−^-cotransporters (KCC) and Cl^−^/HCO_3_^−^-antiporters (Payne et al., 2003; Blaesse et al., 2009; Figure 1), whereas there is currently little evidence for a neuronal Cl^−^-dependent ATPase or Cl^−^ pump (Gerencser and Zhang, 2003). The most important Cl^−^ loader in neurons is NKCC1 (SLC12A2), an ubiquitously expressed Cl^−^ transporter that utilizes the inwardly directed Na^+^ gradient to mediate an electroneutral import of Cl^−^ ions (and K^+^ ions) into cells (Russell, 2000; Virtanen et al., 2020). The main transporter responsible for the low [Cl^−^]_i_ of mature neurons is KCC2 (SLC12A5; Rivera et al., 1999; Lee et al., 2005), which uses the outwardly directed K^+^ gradient to extrude Cl^−^. In addition, the isoforms KCC1, KCC3, and KCC4 were expressed in some neuron populations, but these isoforms can also be found in non-neuronal tissue (Becker et al., 2003). The anion exchanger (AE3) mediates the counter-transport of one Cl^−^ with one HCO_3_^−^, thus leading to Cl^−^ accumulation at physiological pH values (Gonzalez-Islas et al., 2009; Pfeffer et al., 2009). The contribution of Na^+^-dependent Cl^−^/HCO_3_^−^-antiporters, which mediate Cl^−^ extrusion, to neuronal [Cl^−^]_i_ homeostasis is less clear (Huebner and Holthoff, 2013). And finally, Misgeld and coworkers demonstrated that the combination of voltage-dependent Cl^−^ channels with depolarizing E_m_ transients can also lead to an elevated [Cl^−^]_i_ (Titz et al., 2003).

GABA_A_ receptors also have a considerable HCO_3_^−^ permeability (Farrant and Kaila, 2007; Blaesse et al., 2009). The relative HCO_3_^−^ permeability of GABA_A_ receptors is between 0.18 and 0.44 of the Cl^−^ permeability (Bormann et al., 1987; Fatima-Shad and Barry, 1993). Due to the rather positive equilibrium potential for HCO_3_^−^ (E_HCO3_), which is around −10 mV, the HCO_3_^−^ fluxes always add a depolarizing component to the GABAergic current (Rivera et al., 2005; Huebner and Holthoff, 2013). The high E_HCO3_ is a consequence of the low intracellular HCO_3_^−^ concentration ([HCO_3_^−^]_i_), which is on one hand maintained by secondary active HCO_3_^−^ uptake *via* electroneutral and electrogenic Na^+^/HCO_3_^−^ symporters (Sinning et al., 2011; Huebner and Holthoff, 2013). On the other hand, the [HCO_3_^−^]_i_ is directly linked to the intracellular pH (pH_i_) *via* the carbonic anhydrase (Sinning and Hübner, 2013). Thus at physiological pH values between 7.0 and 7.4 (Ruffin et al., 2014), which is maintained by aforementioned Na^+^/HCO_3_^−^ symporters and the Na^+^/H^+^ exchanger (Ruffin et al., 2014), [HCO_3_^−^]_i_ values of ca. 14 mM can be estimated (Lombardi et al., 2019). In consequence, E_GABA_ is typically positive to E_Cl_, albeit the contribution of E_HCO3_ to E_GABA_ becomes smaller with higher [Cl^−^]_i_ (Farrant and Kaila, 2007). During massive GABAergic stimulation, e.g., during epileptic seizures, the stable depolarizing drive of the GABAergic HCO_3_^−^ currents will enhance activity-dependent Cl^−^ uptake and thus directly contributes to the generation of GABAergic excitation under this conditions (Kaila et al., 1997; Ruusuvuori et al., 2004).

## Expression Profile of Cl^−^ Loaders and Cl^−^ Extruders (And Why This Does Not Tell Everything About GABA Actions)

During development the different Cl^−^-transporters are differentially expressed in the nervous system (Blaesse et al., 2009; Huebner and Holthoff, 2013; Kaila et al., 2014; Kirmse et al., 2018). The expression levels of NKCC1 vary significantly between neuronal cell types and between different developmental or functional states of individual neurons (Watanabe and Fukuda, 2015; Virtanen et al., 2020).

Experimental studies show that NKCC1 expression either declines (Plotkin et al., 1997) or increases (Clayton et al., 1998) during development. Therefore, no general statements about the trend of NKCC1 expression level during development could be made (Virtanen et al., 2020). In contrast, the expression of KCC2 has been tightly correlated to the developmental downregulation of [Cl^−^]_i_. Suppression of functional KCC2 expression increases neuronal [Cl^−^]_i_ (Rivera et al., 1999; Pellegrino et al., 2011), whereas enhancing the functional expression of KCC2 during early developmental stages leads to a reduced neuronal [Cl^−^]_i_ (Lee et al., 2005). The expression of KCC2 occurs typically delayed to the expression of NKCC1 (Lu et al., 1999; Rivera et al., 1999; Stein et al., 2004), with the temporal profile of KCC2 expression depending on brain structures (Watanabe and Fukuda, 2015), cortical layers (Li et al., 2002; Shimizu-Okabe et al., 2002), and neuronal cell types (Ikeda et al., 2003; Clarkson and Herbison, 2006). In particular, GABAergic interneurons seem to display systematically more depolarized GABA reversal potentials (E_GABA_) than glutamatergic or principle neurons in the amygdala and neocortex (Martina et al., 2001), the cerebellum (Chavas and Marty, 2003), and in the hippocampus (Elgueta and Bartos, 2019; Otsu et al., 2020). For the hippocampus this has directly been related to a lower KCC2 expression in the GABAergic interneurons (Elgueta and Bartos, 2019). There are also evidences that the expression ratio between NKCC1 and KCC2 can be different in distinct compartments of the same cell (Virtanen et al., 2020). The most striking example is the axon initial segment, in which GABAergic synapses mediate depolarizing responses with putative excitatory effect (Szabadics et al., 2006; Khirug et al., 2008), which has been linked to the delayed shift in the expression ration of Cl^−^ transporter expression in this compartment (Rinetti-Vargas et al., 2017). The observation of a somatodencritic [Cl^−^]_i_ gradient (Kuner and Augustine, 2000; Elgueta and Bartos, 2019) suggest a variable NKCC1/KCC2 expression ratio within dendritic membranes.

A variety of pathophysiological conditions are also linked to massive changes in the [Cl^−^]_i_ -homeostasis (Kaila et al., 2014). It has been shown that depolarizing GABAergic responses and/or an high NKCC1/KCC2 expression ration can be re-attained for example after traumatic (Toyoda et al., 2003; Dzhala et al., 2012) or ischemic (Jaenisch et al., 2010) insults, in peri-tumor regions (Pallud et al., 2014; Campbell et al., 2015) and as cause or consequence of epilepsy (Fujiwara-Tsukamoto et al., 2003; Aronica et al., 2007; Huberfeld et al., 2007; Buchin et al., 2016; Burman et al., 2019; Liu et al., 2020). The putative switch in GABAergic responses from excitation to inhibition related to this alterations can aggravate the clinical consequences of these neuropathologies (Kaila et al., 2014).

However, it should always be kept in mind that both KCC2 and NKCC1 are highly regulated proteins (Russell, 2000; Blaesse et al., 2009; Kaila et al., 2014). For example it has been demonstrated that membrane trafficking and functional expression of KCC2 is tightly controlled by the sonic hedgehog pathway, neuronal-restricted silencing element or the neurotrophin BDNF (Karadsheh and Delpire, 2001; Rivera et al., 2002; Delmotte et al., 2020). In addition, the activity of KCC2 can be modulated by phosphorylation (Wake et al., 2007; Banke and Gegelashvili, 2008) and thereby neurotransmitters can directly interfere with the [Cl^−^]_i_ homeostasis (Banke and Gegelashvili, 2008; Inoue et al., 2012; Yang et al., 2015). Thus the [Cl^−^]_i_ is clearly regulated beyond the expression ratio of Cl^−^ transporters.

The situation is made even more complicated by the fact that [Cl^−^]_i_, is also directly influenced by GABAergic activity *via* Cl^−^ fluxes across GABA_A_ receptors (Wright et al., 2011; Raimondo et al., 2012; Branchereau et al., 2016). This is not only relevant for high, pathophysiological activity patterns, but also for physiological levels of neuronal activity (Gonzalez-Islas et al., 2010; Kolbaev et al., 2011b; Currin et al., 2020). In-silico experiments show that these activity-dependent [Cl^−^]_i_ changes are influenced by the dendritic morphology, membrane properties as well as the kinetics of GABAergic inputs, [Cl^−^]_i_ homeostasis and [HCO_3_^−^]_i_ homeostasis (Doyon et al., 2016a; Mohapatra et al., 2016; Düsterwald et al., 2018; Lombardi et al., 2019). In addition, such activity-dependent [Cl^−^]_i_ transients are augmented by coincident glutamatergic inputs (Halbhuber et al., 2019; Lombardi et al., 2021). Thus the impact of neuronal activity on [Cl^−^]_i_ is probably particularly relevant under *in vivo* situations, where an ongoing “bombardment” with GABAergic and glutamatergic synaptic inputs has been suggested (Steriade, 2001).

In summary, these facts demonstrate that, although an increasing KCC2 expression is correlated to a [Cl^−^]_i_ decline during neuronal development, the precise [Cl^−^]_i_, and thus the GABAergic effects cannot be directly estimated from the general expression level of KCC2. In particular, results from *in vitro* experiments may overestimate the *in vivo* levels of [Cl^−^]_i_ in immature neurons (and underestimate them in adult neurons), because the influence of neuronal activity on [Cl^−^]_i_ is negligible due to the limited activity in the *in vitro* preparations.

## Relation Between [Cl^−^]_I_, GABAergic Membrane Responses and Excitation/Inhibition

The [Cl^−^]_i_ is a main factor that determines EMF_Cl_ and thus the direction and size of Cl^−^ fluxes through the anion-pore of GABA_A_ receptors (Farrant and Kaila, 2007). As mentioned before, HCO_3_^−^ fluxes contribute to GABAergic membrane responses, because GABA_A_ receptors have a considerable HCO_3_^−^ permeability (Farrant and Kaila, 2007). EMF_HCO3_ is directed outwards and therefore HCO_3_^−^ efflux adds a depolarizing component to GABAergic responses. Whereas the HCO_3_^−^ fluxes shift GABA responses in depolarizing direction for low [Cl^−^]_i_, their contribution is relatively small at higher [Cl^−^]_i_ (Farrant and Kaila, 2007). Because of its mostly minor contribution and in order to make the following considerations more concise, HCO_3_^−^-fluxes will not be taken into account in the remainder of this review. Nevertheless, the (small) depolarizing HCO_3_^−^-currents *via* GABA_A_ receptors will slightly enhance the excitatory potency of GABAergic effects.

**Figure 2:**
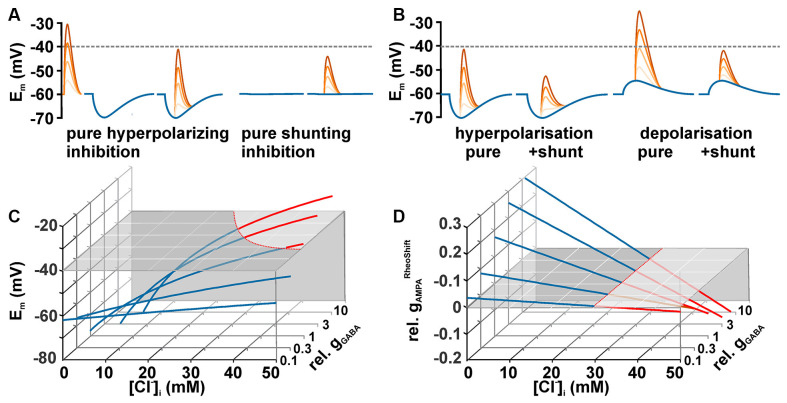
Dependency between [Cl^−^]_i_ and GABAergic actions. **(A)** Schematic diagrams illustrating the two exemplary effects of GABAergic inputs (blue traces) on glutamatergic inputs (red traces) of different intensities. A GABAergic hyperpolarization augments the distance between peak glutamate depolarization and the AP threshold (hyperpolarizing inhibition). At a passive Cl^−^-distribution GABA does not affect E_m_, but the decreased membrane resistivity induced by GABA reduced the peak glutamate depolarization (shunting inhibition). The dashed line represents a hypothetical action potential threshold. **(B)** Schematic diagrams illustrating that the combination of the membrane potential shift with the shunting effect caused by the decreased membrane resistivity augments the effect of a hyperpolarization inhibition (left traces) and can lead to inhibition even at depolarizing GABAergic membrane responses (right traces). **(C)** [Cl^−^]_i_-dependency of the membrane potential (E_m_) calculated for five different GABAergic conductances (g_GABA_, normalized to g_Input_) under stationary conditions (see main text for details). The gray plane represents AP threshold. Note that considerable g_GABA_ in combination with high [Cl^−^]_i_ is needed for a suprathreshold GABAergic depolarization. **(D)** Dependency of g_AMPA_^Rheoshift^ (normalized to g_Input_, ; g_AMPA_^Rheoshift^_ = gAMPA_ in presence of GABA minus _gAMPA_ in absence of GABA) on [Cl^−^]_i_ and g_GABA_. Red traces indicate excitatory and blue traces inhibitory GABAergic effects. Note that g_GABA_^Rheoshift^ becomes negative at identical [Cl^−^]_i_ independent of g_GABA_.

When considering the effect of GABA on neuronal excitability, one should keep in mind that GABA can mediate inhibition by two mechanisms (Farrant and Kaila, 2007): first, by hyperpolarization, which increases the difference between E_m_ and the AP threshold (Figure 2A, left traces), and second by a decreased membrane resistivity upon GABA_A_ receptor activation (Figure 2A, right traces), which shunts excitatory synaptic inputs (Edwards, 1990; Staley and Mody, 1992; Farrant and Kaila, 2007). However, in reality both effects act in parallel (Figure 2B). To predict the effects of GABA on the excitability, it is necessary to delineate how both mechanisms are related to [Cl^−^]_i_. If EMF_Cl^−^_ is negative (i.e., [Cl^−^]_i_ is below the passive distribution) activation of GABA_A_ receptors will induce a Cl^−^ influx and thus hyperpolarize the membrane. Such a membrane hyperpolarization, together with the membrane shunting, will increases the amount of excitatory synaptic currents required to cross the AP threshold. Thus it is obvious that under low [Cl^−^]_i_ conditions GABA_A_ receptors mediate an inhibitory effect on neuronal membranes. It is sometimes considered that at higher [Cl^−^]_i_ GABA receptors mediate an opposite effect, because the depolarizing GABAergic responses shift E_m_ towards AP threshold. However, under this condition the excitatory influence of this depolarizing effect is opposed by the reduction in the membrane resistivity, which in parallel shunts excitatory postsynaptic potentials (Edwards, 1990; Staley and Mody, 1992; Egawa and Fukuda, 2013). Thereby depolarizing GABAergic responses can also reduce excitatory influence and thus mediate inhibition (Figure 2B). The central question arising from these considerations is: Which GABAergic membrane depolarization is required to mediate an excitatory response, i.e., to increase the probability to trigger an AP?

Theoretically, this question can be easily addressed by calculating the membrane depolarization and the shunting effect that are caused by a given GABAergic conductance. The maximal GABAergic depolarization is, in accordance with Ohm’s law, given by the product of the GABAergic current (I_GABA_) and the input resistance (R_Input_). I_GABA_ is given by the product of EMF_Cl_ (when the GABAergic HCO_3_^−^ permeability is neglected) and g_GABA_. A combination of these functions define the relation between g_GABA_ and EMF_Cl_ and can be used to estimate under which conditions GABA responses itself can reach AP threshold and trigger APs (Figure 2C).

However, this situation does not reflect the physiologically relevant situation. More relevant is the question whether subtreshold GABAergic depolarizations can attenuate or augment the excitatory effect of additional excitatory synaptic inputs. In this respect it must be considered that the increase in g_GABA_, which is necessarily linked to the depolarization, also reduces the membrane resistivity and thus shunts excitatory synaptic inputs. The impact of both effects on the excitability can be estimated in a simplified model for E_m_ (which neglects the HCO_3_^−^ conductance of GABA_A_ receptors) as follows:


$$E_{\text{m}}=\frac{RT}{F}*\ln\;\left(\frac{g_{\text{Na}}^{\text{pas}}{\left[{\text{Na}}^{+}\right]}_{\text{e}}+g_{\text{K}}^{\text{pas}}{\left[{\text{K}}^{+}\right]}_{\text{e}}+g_{\text{GABA}}{\left[{\text{Cl}}^{-}\right]}_{\text{i}}+g_{\text{AMPA}}{\left[{\text{Na}}^{+}\right]}_{\text{e}}+g_{\text{AMPA}}{\left[{\text{K}}^{+}\right]}_{\text{e}}}{g_{\text{Na}}^{\text{pas}}{\left[{\text{Na}}^{+}\right]}_{\text{i}}+g_{\text{K}}^{\text{pas}}{\left[{\text{K}}^{+}\right]}_{\text{i}}+g_{\text{GABA}}{\left[{\text{Cl}}^{-}\right]}_{\text{e}}+g_{\text{AMPA}}{\left[{\text{Na}}^{+}\right]}_{\text{i}}+g_{\text{AMPA}}{\left[{\text{K}}^{+}\right]}_{\text{i}}}\right)$$

(with $g_{\text{Na}}^{\text{pas}}$ and $g_{\text{K}}^{\text{pas}}$ as passive membrane conductance for Na^+^ and K^+^, *g*_GABA_ and *g*_AMPA_ as conductance of GABA and AMPA receptors, and [X^+^]_i_ and [X^+^]_e_ as intra- and extracellular concentration of the ion X^+^, respectively). Note that at this moment we use a rather simple model that neglects capacitive currents, the time course of GABAergic and glutamatergic conductances, as well as spatial integration (Gidon and Segev, 2012). Thus this formula describes only the interaction of stationary GABAergic and glutamatergic conductances.

Using this formula, the *g*_Glu_ value leading to a depolarization that equals the AP threshold (*E*_Thr_) can be calculated as follows:


$$g_{\text{Glu}}^{\text{Thr}}=\frac{e^{\text{F}}g_{\text{K}}^{\text{pas}}{\left[{\text{K}}^{+}\right]}_{\text{e}}-g_{\text{K}}^{\text{pas}}{\left[{\text{K}}^{+}\right]}_{\text{i}}-e^{\text{F}}g_{\text{K}}^{\text{pas}}{\left[{\text{Na}}^{+}\right]}_{\text{e}}-g_{\text{K}}^{\text{pas}}{\left[{\text{Na}}^{+}\right]}_{\text{i}}+e^{\text{F}}g_{\text{GABA}}{\left[{\text{Cl}}^{-}\right]}_{\text{i}}-g_{\text{GABA}}{\left[{\text{Cl}}^{-}\right]}_{\text{e}}}{{\left[{\text{Na}}^{+}\right]}_{\text{i}}+{\left[{\text{K}}^{+}\right]}_{\text{i}}-e^{\text{F}}{\left[{\text{K}}^{+}\right]}_{\text{a}}-e^{\text{F}}{\left[{\text{Na}}^{+}\right]}_{\text{a}}}$$

$$with\;e^{\text{F}}=10^{\frac{E_{\text{Thr}}}{-61\text{mv}}}\;(\text{for a given T of }37^{\circ }\text{ C})$$

These threshold *g*_Glu_ values ($g_{\text{Glu}}^{\text{Thr}}$) describe the excitatory conductance required to just reach AP threshold. To quantify the GABAergic effect on the excitability, $g_{\text{Glu}}^{\text{Thr}}$ determined in the absence of GABA (i.e., *g*_GABA_ = 0) is subtracted from $g_{\text{Glu}}^{\text{Thr}}$ determined in the presence of GABA. This value is termed GABAergic rheobase shift ($g_{\text{Glu}}^{\text{Rheoshift}}$). Negative $g_{\text{Glu}}^{\text{Rheoshift}}$ values characterize an excitatory GABAergic action (less *g*_Glu_ is required to induce APs). Intriguingly, GABA mediates an excitatory action, independent of the *g*_GABA_ values, for all [Cl^−^]_i_ ≥ 29.5 mM in the exemplary simulated neuron used for Figure 2D. Note that this [Cl^−^]_i_ corresponds to an *E*_GABA_ that is identical to the AP threshold of −40 mV used in this model. This relation suggests that GABA mediate an excitation whenever *E*_GABA_ is positive to AP threshold. This theoretical suggestion is in line with previous assumptions that GABA mediate an excitatory effect as long as *E*_GABA_ is above AP threshold (Ben-Ari, 2002; Owens and Kriegstein, 2002) and it was replicated in patch-clamp experiments (Kolbaev et al., 2011a).

However, as already mentioned the previous considerations are clearly an oversimplification as they: (i) neglect additional voltage gated conductances that contribute to excitability (Valeeva et al., 2010); and (ii) represent only stationary conductances in a quasi one-dimensional situation ignoring the consequences of the temporal relation between GABA and glutamatergic inputs (Gao et al., 1998; Gulledge and Stuart, 2003) and the complex neuronal topologies (Jadi et al., 2012; Spruston et al., 2016) on spatiotemporal properties of GABAergic inhibition/excitation.

**Figure 3:**
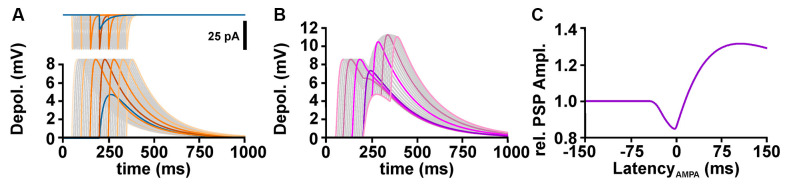
Temporal profile of GABAergic shunting and GABAergic depolarizing effects on excitatory glutamatergic inputs. **(A)** The upper traces illustrate GABAergic (blue line) and glutamatergic (orange and gray lines) currents provided at latencies between −150 and +150 ms. The lower traces illustrate the postsynaptic potentials (PSPs) evoked by these currents. Note that the PSPs outlast the synaptic currents. **(B)** Compound PSPs induced by the co-stimulation of GABA and glutamate synapses, with the glutamatergic inputs provided at latencies between −150 and +150 ms. **(C)** Peak amplitude of compound PSP, normalized to the glutamatergic PSP in the absence of GABA, plotted against the latency between AMPA and GABA stimuli, as shown in **(B)**. Note that the compound PSP amplitude drops if glutamatergic synapses are activated within a narrow interval around coincident stimulation, but increases when AMPA receptors are stimulated several ms after the GABA input.

To understand the influence of temporal relation between GABA and glutamatergic inputs on the excitability, it is important to consider that the GABAergic membrane depolarization outlasts the GABAergic conductance increase (Figure 3A). The amplitude of a glutamatergic excitatory postsynaptic potential (ePSP) drops by shunting effects when it was evoked in synchrony to the GABAergic input. However, when the glutamatergic input was stimulated during the late phase of the GABAergic depolarization (when the GABAergic conductance ceases, but the GABAergic depolarization is still present), temporal summation lead to an increased peak voltage of the compound postsynaptic potential (PSP; Figure 3B). Thereby, GABA can mediate a substantial inhibitory effect on synchronously occurring glutamatergic inputs, while the longer lasting depolarization can enhance the amplitude of delayed ePSPs and thus mediate excitation (Figure 3C). Such a temporal shunting-to-excitation sequence has already been shown *in vitro* (Gao et al., 1998; Gulledge and Stuart, 2003; Bracci and Panzeri, 2006). This finding also implies that for excitatory inputs that occur with a substantial delay after the GABA input mainly the GABAergic depolarization is effective and thus an excitatory effect can be imposed whenever E_GABA_ is positive to RMP. In consequence, GABA may more probably have an excitatory effect when GABA inputs are not temporally correlated to glutamatergic inputs, e.g., for tonic GABAergic inhibition (Song et al., 2011; Kolbaev et al., 2012). On the other hand, when GABA and glutamate inputs are temporally highly correlated, e.g., at feedback, feedforward, or lateral inhibition, GABA mediates a reliable inhibition as long as E_GABA_ is below AP threshold.

**Figure 4:**
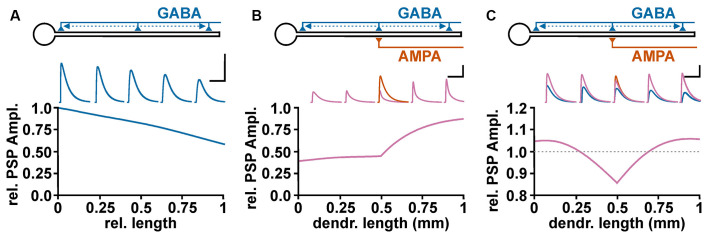
Spatial profile of GABAergic depolarization and GABAergic shunting effects on excitatory glutamatergic inputs. **(A)** Relative amplitude of GABAergic PSPs, as measured at the soma, upon activation of a depolarizing GABA synapse (E_GABA_ = −52 mV) at different dendritic positions. The voltage traces above graph illustrate GABAergic PSPs at 0%, 25%, 50%, 75%, and 100% of the dendritic length. Scale bar in **(A–C)** is 5 mV/500 ms. **(B)** Profile of the GABAergic shunting effect on glutamatergic inputs, calculated by normalizing the amplitude of the compound PSPs obtained in the presence of GABA (purple traces) to the EPSC amplitude obtained in the absence of GABA inputs (orange trace). In these experiments the shunting effect was isolated by maintaining E_GABA_ at resting membrane potential. Note that GABA synapses located proximally to the AMPA synapse (“on-path”) mediate a stable shunting effect, while for GABA synapses distal to the AMPA synapse (“off-path”) the shunting effect declines rather fast. **(C)** Effect of a depolarizing GABAergic input (E_GABA_ = −52 mV) at different positions along the dendrite on the peak compound PSP amplitude during co-stimulation The blue traces represent purely GABAergic PSPs, the orange trace the glutamatergic PSP, and the purple traces the compound PSPs upon co-activation of AMPA and GABA synapses. Note that GABA inputs mediate an inhibitory effect when co-localized with the AMPA synapse, while at more distant on-path and off-path synapses an excitatory effect is observed.

To address the role of complex neuronal topologies on the GABAergic effect, it must be considered: (i) that GABA_A_ receptor activation influences the length and time constant of membranes and (ii) that the GABAergic effect depends on the spatial relation between the GABAergic and the glutamatergic inputs. A simple NEURON-based in-silico simulation demonstrate that a GABAergic PSPs showed the typical decline in the amplitude with increasing dendritic distance (Figure 4A), which reflects the length constant within a linear neuronal structure (Rall, 1989). On the other hand, the membrane shunting effect demonstrated a more complex behavior (Gulledge and Stuart, 2003; Gidon and Segev, 2012). If the GABA synapse is located between the soma and the excitatory glutamatergic input (“on-path”, Gidon and Segev, 2012) a stable attenuation of the ePSP amplitude occurs (Figure 4B). But when the GABA synapse is distal to the AMPA synapse (“off-path”), the shunting declines rather fast (Figure 4B). To estimate whether GABA has an excitatory or inhibitory influence, the interaction between the wide spreading depolarizing effect (Figure 4A) and the complex spatial profile of shunting inhibition (Figure 4B) is fundamental. The co-stimulation of a depolarizing GABA synapse which is co-localized to an AMPA synapse mediate an inhibitory effect (Figure 4C), suggesting a dominance of the shunting effect. With increasing distance between the GABA and the AMPA synapse the shunting effect was attenuated and the excitatory potential of the GABAergic depolarization dominates, leading to an excitatory effect of the co-stimulation (Figure 4C). Thus even under mild depolarizing conditions found in dendrites of mature cortical pyramidal cells (Kuner and Augustine, 2000) GABAergic stimulation in the remote dendritic compartment can mediate an excitatory response (Gulledge and Stuart, 2003).

Of note, these findings have some implication for the inhibition mediated by GABA receptors. The typical perisomatic GABAergic inputs of parvalbumin-positive basket interneurons (Freund, 2003; Elgueta and Bartos, 2019) will mediate a stable inhibitory effect, even at depolarizing GABAergic responses, since they can effectively shunt ePSPs. In contrast, for GABAergic synapses located in the dendritic periphery, e.g., from hippocampal O-LM interneurons (Somogyi and Klausberger, 2005) or neocortical Martinotti interneurons (Ascoli et al., 2008; Gidon and Segev, 2012), depolarizing GABA responses can more easily mediate an excitatory effect on glutamatergic inputs from distant sites in the dendrite.

In summary, the action of GABA did not only depend on the ratio between E_GABA_ and the AP threshold, but also on the spatiotemporal relation between the GABAergic and glutamatergic inputs. Thus, under physiological conditions depolarizing GABAergic inputs can mediate in the same cell excitation as well as inhibition, depending on the exact spatiotemporal relation between both inputs. Thus it is difficult or even impossible to make general predictions for a global effect of depolarizing GABAergic responses. However, from the published observations one can presume: (i) that at sufficiently high E_GABA_ above the AP threshold reliable excitation is mediated by GABA_A_ receptors; (ii) that at intermediate E_GABA_ levels GABA mediates a dominant inhibitory effect for spatially and temporally correlated inputs; and (iii) that the effect of GABA on delayed or spatially separated inputs can be excitatory under this conditions. Thus the typical GABAergic feedforward or feedback loops with perisomatic terminals will already mediate inhibition, even at higher [Cl^−^]_I_ that are typical during development (Farrant and Kaila, 2007; Blaesse et al., 2009). In addition, these synapses will be rather resistant to activity-dependent [Cl^−^]_i_ increases (Wright et al., 2011; Doyon et al., 2016b; Lombardi et al., 2018). In contrast, other modes of GABAergic mechanisms are more prone to mediate an excitatory influence at rather moderate [Cl^−^]_i_ increases.

## Examples for Excitatory and Inhibitory GABAergic Effects in The Immature CNS

With all of the information provided above, one of the major questions remaining is, of course, whether the depolarizing GABA_A_ receptor-mediated responses in the immature CNS (Ben Ari et al., 2012) have a net excitatory or inhibitory effect.

Several *in vitro* studies demonstrate that activation of GABA_A_ receptors can mediate excitatory inputs in immature neurons. For example, it was demonstrated that hippocampal giant depolarizations critically depend on tonic depolarizing GABAergic currents (Ben-Ari et al., 1989; Sipila et al., 2005). Gramicidin-perforated or cell-attached patch-clamp experiments, which both did not artificially alter [Cl^−^]_i_ and thus allow estimating the physiological GABA responses, demonstrated suprathreshold GABAergic responses in neocortical (Dammerman et al., 2000; Hanganu et al., 2002; Rheims et al., 2008; Sava et al., 2014), hippocampal (Khazipov et al., 1997; Leinekugel et al., 1997; Sauer and Bartos, 2010; Valeeva et al., 2013), and hypothalamic neurons (Wang et al., 2001). And finally, optogenetic activation of GABAergic interneurons *in vitro* increases the frequency of neocortical and hippocampal EPSCs (Valeeva et al., 2016), as well as synchronous network activity (Flossmann et al., 2019), demonstrating a direct excitatory effect of GABA in neuronal networks. Excitatory GABAergic actions have also been found in mature neurons for distant off-path GABAergic inputs (Gulledge and Stuart, 2003).

In contrast to these reports of excitatory GABAergic actions, several *in vitro* studies also demonstrate that GABAergic stimulation can mediate inhibition already in early postnatal neurons (Agmon et al., 1996; Khalilov et al., 1999; Lamsa et al., 2000). The frequent observations that inhibition of GABA_A_ receptors provoke epileptiform discharges in the immature CNS (Khalilov et al., 1999; Wells et al., 2000; Richter et al., 2010; Kolbaev et al., 2012; Sharopov et al., 2019) also suggest that GABA may mediate a net inhibitory effect in immature hippocampal and neocortical networks. However, depolarizing GABAergic responses has been suggested to significantly contribute to epilepsy in the immature CNS (Dzhala and Staley, 2003; Dzhala et al., 2005; Khalilov et al., 2005; Nardou et al., 2009, 2013). This discrepancy most probably reflects the complexity of functional consequences of depolarizing GABAergic responses. Notably, *in vitro* experiments demonstrated that weak GABAergic stimulation can promote excitation, whereas stronger GABAergic currents mediate inhibition (Khalilov et al., 1999; Winkler et al., 2019), indicating that the balance between GABAergic depolarization and shunt determines the net effect. In line with the aforementioned spatiotemporal dependency of GABAergic effects, it has been observed in the immature hippocampus that synaptic GABA_A_ receptors mediate an anticonvulsive and tonic GABA_A_ receptors a proconvulsive effect (Kolbaev et al., 2012). In summary, these experiments promote the view that it depends, in addition to the [Cl^−^]_i_, on the properties and mode of GABAergic stimulation whether GABA has a pro- or anticonvulsive effect.

However, the *in vitro* experiments summarized above represent fairly artificial conditions that may severely interfere with [Cl^−^]_i_ homeostasis. The slicing procedures used for the generation of most *in vitro* preparation represent a traumatic insult, which alters the expression and function of NKCC1 and/or KCC2 and led to an increased [Cl^−^]_i_ in many neurons within such preparations (Dzhala et al., 2012). In addition, neuronal activity, and thus most probably also the frequency of GABAergic inputs, is massively reduced in most *in vitro* preparations (Steriade, 2001). However, the frequency of GABAergic inputs massively influences [Cl^−^]_i_ and lead in immature neurons to a reduction of their high [Cl^−^]_i_ (Kolbaev et al., 2011b; Wright et al., 2011; Lombardi et al., 2018). Thus, *in vitro* condition may systematically overestimate the excitatory capacity of GABA_A_ receptors. Thus it is essential that the effect of GABA in immature nervous systems must also be investigated under *in vivo* conditions.

Seminal *in vivo* experiments addressing the functional responses of GABA on cortical neurons during early developmental stages demonstrated that exogenously applied GABA indeed mediates depolarizing membrane responses, but that these responses reduce neuronal activity in the developing neocortex (Kirmse et al., 2015). In line with these results, optogenetic activation of GABAergic interneurons *in vivo* decreases the frequency of neocortical and hippocampal EPSCs already at early developmental stages (Valeeva et al., 2016), demonstrating a direct inhibitory effect of GABA on neuronal networks. Interestingly, this *in vivo* result is opposing the observation made in the same study under *in vitro* conditions, emphasizing the limitations of conclusions drawn from *in vitro* experiments. Also the observations that the GABA antagonist gabazine enhances the frequency of spindle bust oscillation in the early postnatal neocortex *in vivo* (Minlebaev et al., 2007), and that GABAergic agonists attenuate epileptiform activity *in vivo* (Isaev et al., 2005) already suggest a putative inhibitory role of GABA at this developmental stage.

On the other hand, recent studies demonstrated that activation of GABAergic interneurons *in vivo* can also enhance network activity in the immature hippocampus, suggesting that also under *in vivo* conditions GABA may exert an excitatory effect in this region. Using DREADD as well as optogenetic approaches, it was demonstrated that activation of GABAergic interneurons enhances and inhibition of GABAergic interneurons suppresses network activity in hippocampus of non-anesthetized 3 day old mice pups (Murata and Colonnese, 2020). This effect reversed to GABAergic inhibition already at the 7th postnatal day (Murata and Colonnese, 2020). Comparable results are observed when depolarizing GABAergic responses during early development are minimized by a conditional NKCC1 knockout in pyramidal neurons. In these animals the spontaneous correlated network activity in the hippocampus was attenuated (Graf et al., 2021), suggesting a putative excitatory effect of depolarizing GABAergic responses in the immature hippocampus. However, in line with the previous *in vivo* studies on neocortical areas these two *in vivo* study demonstrated for the visual cortex that activation of GABAergic interneurons mediates inhibition already at the 3rd postnatal day (Murata and Colonnese, 2020) and that a conditional NKCC1 knockout in pyramidal neurons of the visual cortex has no effect on the typical network activity (Graf et al., 2021).

## Conclusion

While it is obvious that the effect of GABA_A_ receptor activation critically depends on the [Cl^−^]_i_, and thus on the expression and function of Cl^−^ transporters, theoretical consideration and many experimental findings indicate that the effect of GABA on the excitability cannot reliably be predicted only from the expression ratio of Cl^−^ transporters or the [Cl^−^]_i_. Several additional parameters determine whether GABA mediate excitation or inhibition at a given [Cl^−^]_i_. Recent experimental evidences suggest that GABA probably mediates inhibition already in the immature cortex, whereas it may contribute to excitation in the immature hippocampus. However, these experiments can, of course, not predict GABAergic effects during fetal stages or in brain structures that have not been investigated yet.

## Author Contributions

WK conceptualized and wrote this manuscript.

## Conflict of Interest

The author declares that the research was conducted in the absence of any commercial or financial relationships that could be construed as a potential conflict of interest.

## Publisher’s Note

All claims expressed in this article are solely those of the authors and do not necessarily represent those of their affiliated organizations, or those of the publisher, the editors and the reviewers. Any product that may be evaluated in this article, or claim that may be made by its manufacturer, is not guaranteed or endorsed by the publisher.
